# Inhibition of Diacylglycerol–Sensitive TRPC Channels by Synthetic and Natural Steroids

**DOI:** 10.1371/journal.pone.0035393

**Published:** 2012-04-17

**Authors:** Susanne Miehe, Peter Crause, Thorsten Schmidt, Matthias Löhn, Heinz-Werner Kleemann, Thomas Licher, Werner Dittrich, Hartmut Rütten, Carsten Strübing

**Affiliations:** Sanofi-Aventis Deutschland GmbH, Research and Development, Frankfurt am Main, Germany; Harvard Medical School, United States of America

## Abstract

TRPC channels are a family of nonselective cation channels that regulate ion homeostasis and intracellular Ca^2+^ signaling in numerous cell types. Important physiological functions such as vasoregulation, neuronal growth, and pheromone recognition have been assigned to this class of ion channels. Despite their physiological relevance, few selective pharmacological tools are available to study TRPC channel function. We, therefore, screened a selection of pharmacologically active compounds for TRPC modulating activity. We found that the synthetic gestagen norgestimate inhibited diacylglycerol-sensitive TRPC3 and TRPC6 with IC_50_s of 3–5 µM, while half-maximal inhibition of TRPC5 required significantly higher compound concentrations (>10 µM). Norgestimate blocked TRPC-mediated vasopressin-induced cation currents in A7r5 smooth muscle cells and caused vasorelaxation of isolated rat aorta, indicating that norgestimate could be an interesting tool for the investigation of TRP channel function in native cells and tissues. The steroid hormone progesterone, which is structurally related to norgestimate, also inhibited TRPC channel activity with IC_50_s ranging from 6 to 18 µM but showed little subtype selectivity. Thus, TRPC channel inhibition by high gestational levels of progesterone may contribute to the physiological decrease of uterine contractility and immunosuppression during pregnancy.

## Introduction

Transient receptor potential canonical (TRPC) channels belong to the TRP cation channel superfamily. Seven TRPC channels, TRPC1–7, have been found in rodents. They all conduct Ca^2+^ in addition to monovalent cations and can be activated via membrane receptors linked to phospholipase C signaling. Downstream of phospholipase C the activation mechanisms of TRPC channels are not well defined but one subclass, namely TRPC3, −6 and −7, as well as the structurally more distinct TRPC2 respond to the phospholipase C hydrolysis product diacylglycerol. The remaining family members, however, are diacylglycerol-insensitive (reviewed in [1]–[3]).

Genetic models have been instrumental in defining the physiological roles of TRPC channels. The involvement of TRPC4 and TRPC6 in vasoregulation [4], [5], TRPC1 in muscle function [6], [7], TRPC2 in pheromone signaling [8], [9], TRPC3 in motor coordination [10], [11], and most recently of TRPC5 [12] in innate fear responses has been elucidated using knockout mice.

Despite the substantial progress made, important questions regarding TRPC channel function and regulation remain, and it would be highly desirable to verify and extent studies in genetically modified mice by pharmacological means in non-engineered animals. Unfortunately, such experiments have proved difficult due to the lack of specific compounds that modulate TRPC channels. Tools such as SKF96365 [13], [14], KB-R7943 [15] or BTP-2 [16] do not discriminate between TRPC homologs and also alter the activity of other ion channels or transporters [14], [17]. Only recently the first subtype –specific TRPC inhibitor was discovered. This compound, the pyrazole Pyr3, inhibited TRPC3 with a half maximal inhibitory concentration (IC_50_) of 0.7 µM without having effects on other TRPC channels. Using Pyr3 an involvement of TRPC3 in the development of cardiac hypertrophy could be demonstrated *in vivo* [18].

To enrich the repertoire of pharmacological tool compounds for TRPC channels and to identify new natural channel modulators, we screened a library of pharmacologically and biologically active compounds for their effects on TRPC6-mediated Ca^2+^ entry. We found that norgestimate, a synthetic steroid and active ingredient of certain contraceptives, preferentially inhibited TRPC3 and −6 channels at low micromolar concentrations. The endogenous gestagen progesterone also inhibited TRPC channels but showed little subtype selectivity.

Interestingly, a recent study by Majeed et al. [19] demonstrated inhibition of TRPC5 by neurosteroids including pregnenolone sulphate and progesterone. Our data now show that steroid modulation is a common feature of TRPC family members and reveal structural determinants of selective TRPC channel inhibition by progestins.

Hence, TRPC channels may contribute to diverse steroid actions ranging from progesterone-induced vascular remodelling and decrease in uterine contractility during pregnancy to cardiovascular side-effects of oral contraceptives.

## Materials and Methods

### Cell culture and cell line generation

Cells were grown at 37°C in a humidified atmosphere (5% or 7% CO_2_) under standard cell culture conditions. Stable human embryonic kidney (HEK) cell lines expressing recombinant mTRPC4ß (GenBank accession number AAC05178); mTRPC5 (GenBank accession number NM_009428) or hTRPC6 (GenBank accession number AF080394) under the control of a tetracycline-inducible promoter were generated using the Flp-In T-Rex (FITR) system (Invitrogen, Karlsruhe, Germany). TRPC4/5/6 HEK-FITR cells were maintained in Dulbecco's modified eagle medium (DMEM, with glutaMAX I, 4.5 g/l glucose and 110 mg/ml sodium pyruvate) supplemented with 10% (v/v) fetal bovine serum (Biochrom, Berlin, Germany), 1 mM glutamine, 1 mM MEM sodium pyruvate, 40 µg/ml hygromycin (50 µg/ml for mTRPC5 HEK-FITR cells), and 15 µg/ml blasticidine HCl. Channel expression was induced by supplementing the growth medium for 18–24 h with 1 µg/ml doxycycline.

hTRPC3 (GenBank accession number NM_003305) was stably expressed in chinese hamster ovary (CHO) cells using a proprietary high expression vector (Steinbeis –Transferzentrum für Angewandte Biologische Chemie, Mannheim, Germany).

For measurement of norgestimate effects on adrenoceptor signaling a CHO cell line stably expressing human α_1A_-receptors (GenBank accession number NM_000680.2) under control of a CMV promoter was used. CHO cells were kept in HAM's F-12 medium (with glutaMAX I) supplemented with 10% (v/v) fetal bovine serum (Biochrom), 1 mM glutamine, and 0.6 mg/ml geneticin.

A7r5 cells (ATCC, Rockville, USA) were maintained in DMEM (with glutaMAX I, 4.5 g/l glucose and 110 mg/ml sodium pyruvate), supplemented with 10% (v/v) fetal bovine serum (PAA, Pasching, Austria).

### Measurement of intracellular calcium concentration ([Ca^2+^]_i_)

#### Fluo-4 measurements

Cells grown to an almost confluent monolayer on black poly-D-lysine coated 96-well plates (Greiner, Frickenhausen, Germany) were washed with standard extracellular solution (140 mM NaCl, 1 mM MgCl_2_, 5.4 mM KCl, 2 mM CaCl_2_, 10 mM HEPES, 10 mM glucose, pH 7.35) and stained (30 min, room temperature) with dye solution (2 µM fluo-4 AM, 0.02% pluronic F127, 0.1% bovine serum albumin in standard extracellular solution). Cells were washed and either incubated with standard extracellular solution only or with different concentrations of test compounds for 10 min. Fluo-4 fluorescence was excited at 488 nm with an argon laser and measured using a fluorometric imaging plate reader (Molecular Devices, Sunnyvale, USA). All fluorometric measurements were performed at room temperature.

Ca^2+^ entry into TRPC3 CHO and TRPC6 HEK-FITR cells was elicited by application of the diacylglycerol analog 1-oleoyl-2-acetyl-sn-glycerol (OAG). For calculation of norgestimate- and progesterone-induced inhibition of TRPC3- and TRPC6-mediated Ca^2+^ entry fluorescence values were plotted versus time and the area under the curve was considered as a measure of Ca^2+^ influx. Baseline fluorescence in non-OAG-stimulated cells and OAG-stimulated Ca^2+^ influx into control cells not treated with inhibitors were defined as 0% and 100% influx, respectively.

TRPC4- and TRPC5-mediated Ca^2+^ influx was stimulated by application of trypsin that activates endogenous G_q_-coupled protease activated receptors in HEK cells [20]. As trypsin, via phospholipase C activation, causes Ca^2+^ release from internal stores (PI response) and subsequent Ca^2+^ entry through native store-operated channels, these components had to be subtracted from the total fluorescence signal in order to determine TRPC-mediated Ca^2+^ entry. For this purpose trypsin-induced Ca^2+^ responses in non-induced TRPC4- and TRPC5 HEK-FITR cells lacking functional expression of the TRPC channels were measured in parallel to responses in induced cells and were subtracted from the fluorescence traces. Compound effects were then calculated from the area under the curve and normalized to the Ca^2+^ influx into trypsin-stimulated, non-compound-treated cells.

[Ca^2+^]_i_ measurements in CHO cells stably expressing human α_1A_ –receptors were performed essentially as described for HEK-FITR cells. The α_1_- agonist phenylephrine was used for receptor stimulation.

#### Fura-2 measurements

Cells grown on poly-L-lysine-coated 24-mm glass coverslips were loaded in cell culture medium supplemented with 2 µM fura-2 AM (30 min, 37°C) and subsequently allowed to de-esterify in standard extracellular solution (15 min, 37°C). Changes in [Ca^2+^]_i_ were measured using a monochromator-based imaging system (T.I.L.L. Photonics, Gräfelfing, Germany) mounted on an inverted Axiovert 200 microscope (Zeiss, Göttingen, Germany). Fluorescence was excited alternating at 340 nm and 380 nm, long-pass filtered at 440 nm and captured at 2 s intervals. The 340/380 nm excitation ratio of selected cell areas was calculated with T.I.L.L. vision 4.0 software (T.I.L.L. Photonics) after correction for background fluorescence.

### Electrophysiological techniques

The whole-cell patch clamp technique [21] was employed to measure ion currents from single cells. Heat-polished patch pipettes with resistances of 2–4 MΩ were pulled from borosilicate glass capillaries (Hilgenberg, Malsfeld, Germany) using a DMZ-Universal puller (Zeitz-Instruments, Munich, Germany) and filled with standard intracellular solution containing: 120 mM CsOH, 120 mM gluconic acid, 2 mM MgCl_2_, 3 mM CaCl_2_, 5 mM Cs_4_-BAPTA, 10 mM HEPES (pH 7.4 adjusted with gluconic acid). For agonist-independent stimulation of TRPC currents the intracellular solution was supplemented with 30 µM AlCl_3_ and 10 mM NaF and cells were infused with AlF_4_^−^ through the patch pipette. AlF_4_^−^ is a non-specific activator of heterotrimeric G proteins that consequently leads to increased phospholipase C activity and TRPC channel activation.

The extracellular solution for recording of TRPC currents contained: 140 mM NaCl, 5.4 mM KCl, 2 mM CaCl_2_, 1 mM MgCl_2_, 10 mM glucose, 10 mM HEPES (pH 7.4 adjusted with NaOH).

For measurement of [Arg^8^]-vasopressin (AVP) – induced currents in A7r5 cells the Ca^2+^ concentration of the extracellular solution was lowered to 0.2 mM.

Cells grown on poly-L-lysine-coated coverslips were continuously superfused with extracellular solution and substances were applied using an ALA BPS-8 perfusion system (ALA Scientific Instruments, Westbury, USA). Whole-cell recordings were performed with an EPC-10 amplifier and Pulse software (HEKA, Lambrecht, Germany). Cells were held at −70 mV, and current-voltage (I–V) relationships were routinely measured every 2 or 3 s by applying voltage ramps (180 ms) from −100 mV to +80 mV. Data was acquired at 6.67 kHz and filtered with 2.22 kHz. After establishing the whole cell configuration the series resistance was usually <10 MΩ and was compensated 50–70%. All experiments were performed at room temperature.

### Ethics statement

Animals were used for organ retrieval only and were sacrificed according to Sanofi-Aventis Ethical Committee guidelines and to the Guide for the Care and Use of Laboratory Animals published by the National Institutes of Health. Sanofi-Aventis is an authorized institution to house and handle laboratory animals according §11 German Animal Welfare Act, and has a nominated animal welfare officer. Sanofi-Aventis is also AAALAC (Association for Assessment and Accreditation of Laboratory Animal Care International) accredited. The competent authority (Regierungspräsidium Darmstadt) has been notified of the procedure under protocol number T04-01/102.

### In vitro vascular function

Adult male Wistar-Unilever rats (8–11 weeks old; Harlan Winkelmann, Borchen, Germany) were sacrificed by decapitation. Thoracic aortas were excised quickly, transferred to cold (4°C), carbogen (95% O_2_ and 5% CO_2_) bubbled physiological salt solution (119 mM NaCl, 4.7 mM KCl, 1.2 KH_2_PO_4_, 1.2 mM MgSO_4_, 1.6 mM CaCl_2_, 25 mM NaHCO_3_, 11 mM glucose) and rinsed. After removal of connective tissue and perivascular fat aortas were dissected in 5 mm rings, connected to force transducers and equilibrated in carbogen bubbled physiological salt solution at 37°C for 15 min. Aortic rings were set at 1000 mg passive tension and contractile forces were then measured isometrically using standard bath procedures as described earlier [22]. Vessels strongly contracting after application of 60 mM KCl were defined as intact and used for further experiments. To minimize the contribution of endothelium-derived nitric oxide to compound-induced force changes experiments were performed in the presence of the nitric oxide synthase inhibitor nitro-L-arginine methyl ester (L-NAME).

### Materials

DMEM, HAM's F12, glutamine, MEM sodium pyruvate, hygromycin B, blasticidine HCl, geneticin, Fura-2 AM, Fluo-4 AM, Pluronic F127, and Cs_4_-BAPTA were from Invitrogen, MgCl_2_ and MgSO_4_ from Merck (Darmstadt, Germany), doxycycline from BD Biosciences (Heidelberg, Germany), OAG from Avanti Polar Lipids Inc. (Alabaster, USA), and norgestimate from ChemPacific Corporation (Baltimore, USA). All other chemicals were from Sigma-Aldrich (Munich, Germany).

### Statistics

For statistical analysis, analysis of variance was performed with Origin 6.0 software (Microcal Software Inc., Northampton, USA). P values less than 0.05 were considered as statistically significant. IC_50_ were calculated with SigmaPlot (Systat software, San Jose, USA) using the sigmoidal Hill-model: f = 100 (C^n^/(IC_50_^n^+C^n^)); where f is the inhibition, C is the applied drug concentration and n is the Hill coefficient. The half maximal effective concentration (EC_50_) of norgestimate-induced vasorelaxation was calculated analogously with R = y_0_+y_1_C^n^/(EC_50_^n^+C^n^); where R is relaxation, y_o_ and y_1_ are constants, C is the applied drug concentration and n is the Hill coefficient. Averaged data is expressed as means ± SEM.

## Results

**Figure 1:**
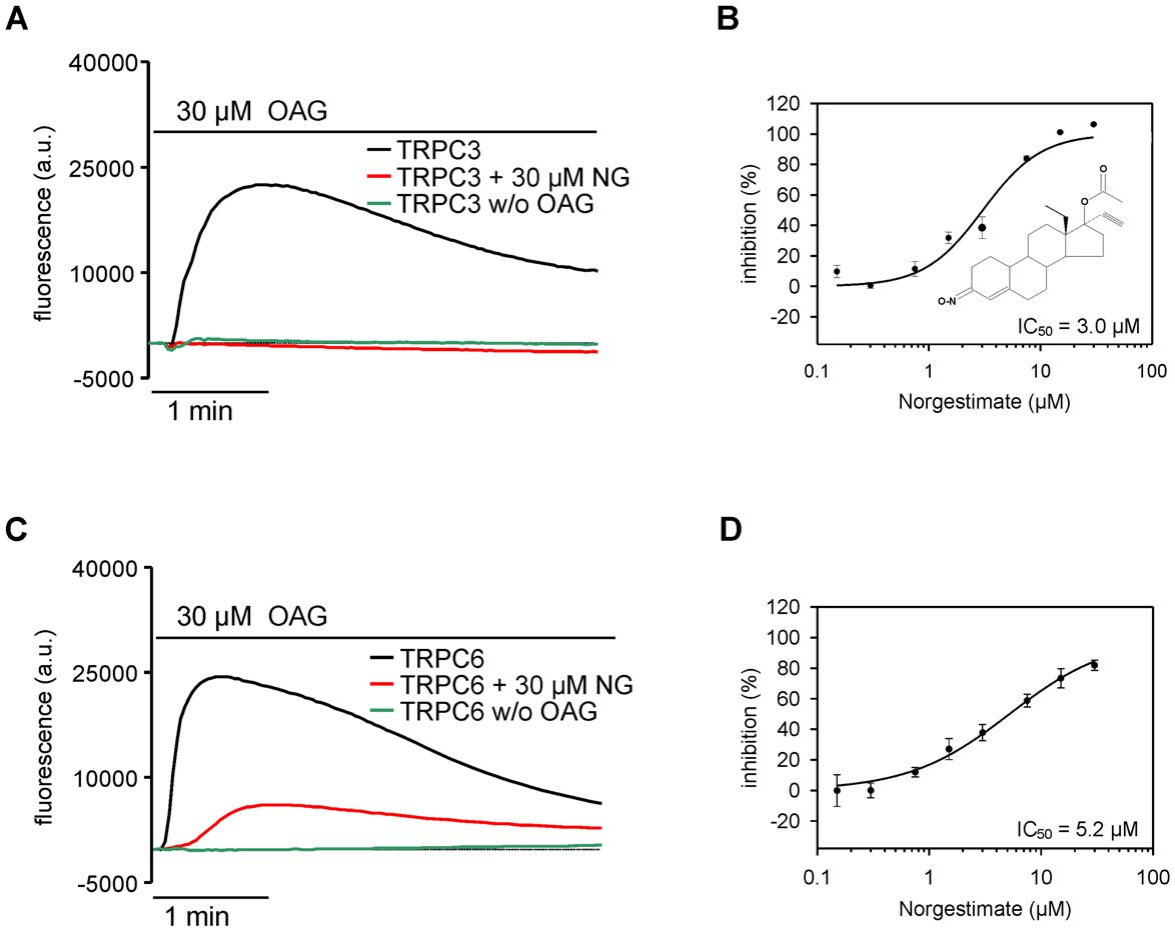
Norgestimate inhibits TRPC3- and TRPC6-mediated Ca^2+^ influx. OAG-induced changes of [Ca^2+^]_i_ in fluo-4-loaded TRPC3 CHO cells (**A**) and TRPC6 HEK-FITR cells (**C**) were measured in 96-well plates using a fluorometric imaging plate reader. Pre-incubation of cells with 30 µM norgestimate (NG) significantly reduced TRPC3 and −6 mediated Ca^2+^ entry. Representative fluorescence traces are shown. Concentration-response curves for inhibition of TRPC3 (**B**) and TRPC6 (**D**) by norgestimate were derived from the area under the fluorescence curves for each given concentration. The solid lines represent the best fit of the data to the Hill model with slopes of n = 1.67 (TRPC3) and n = 0.97 (TRPC6). Means ± SEM of 3 wells (**B**) or 4 wells (**D**) are shown. The chemical structure of norgestimate is illustrated in **B.**

In order to discover compounds that modulate TRPC activity we developed fluorescence-based assays that allowed to measure TRPC-mediated Ca^2+^ entry using fluorometric imaging plate reader technology. Screening a selection of pharmacologically and biologically active compounds using these assays identified norgestimate as a potent TRPC3 and −6 channel inhibitor. Norgestimate blocked OAG-induced Ca^2+^-entry in TRPC3 and TRPC6 expressing cells with similar potency. IC_50_s amounted to 3.0 and 5.2 µM, respectively (**Figure 1**).

**Figure 2:**
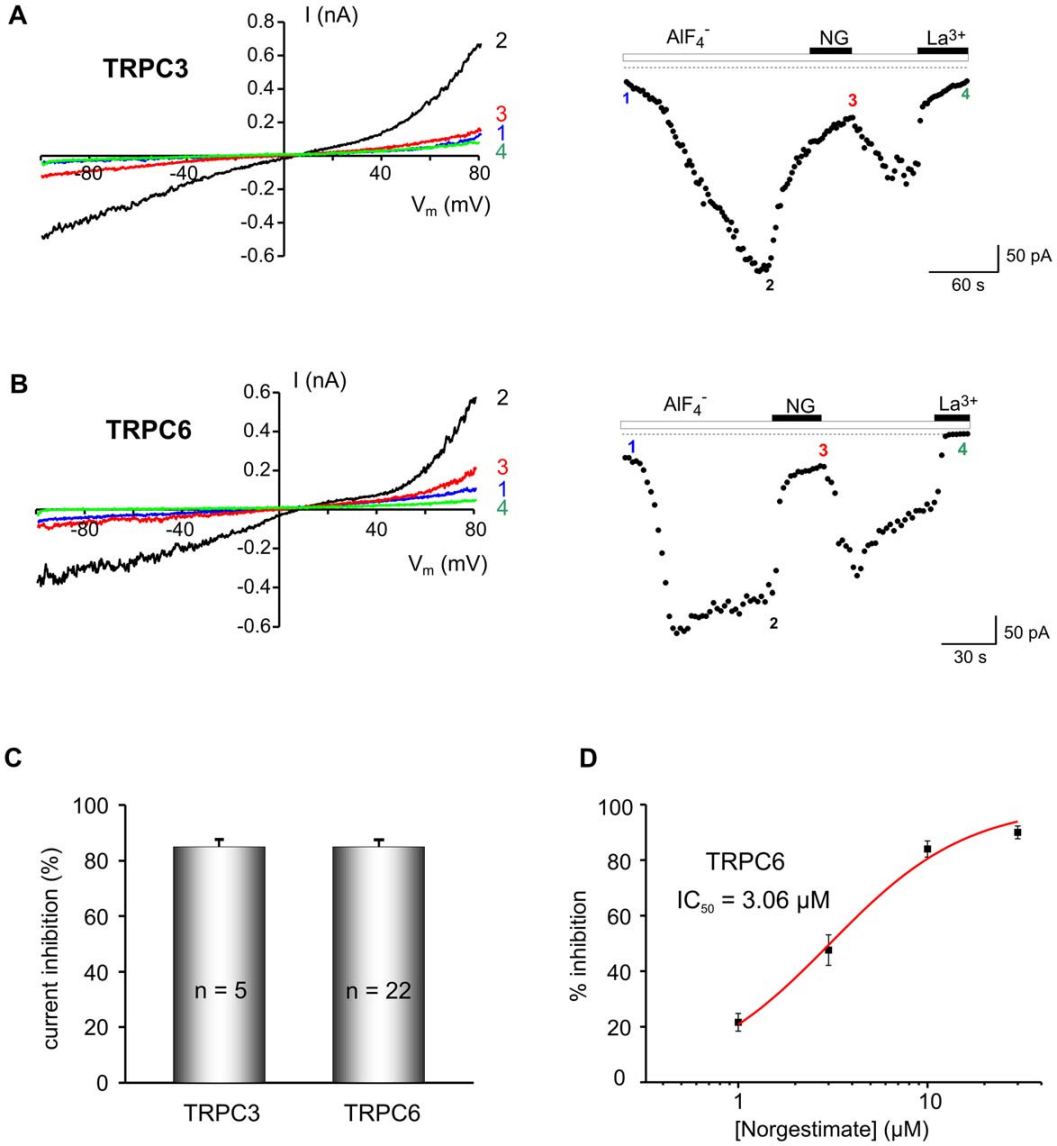
Potent inhibition of TRPC3- and TRPC6-mediated currents by norgestimate. Effect of 10 µM norgestimate (NG) on whole-cell currents evoked by AlF_4_^−^ infusion into TRPC3 (**A**) and TRPC6 (**B**) expressing cells. Current-voltage (I–V) relationships (left panels) and time course of currents recorded at −70 mV (right panels) are shown. For measurement of I–V curves voltage ramps from −100 to +80 mV were applied at the time points indicated. Background currents were isolated by blocking of TRPC3 and −6 with 100 µM La^3+^. Comparison of the mean inhibition of TRPC3 and −6 by 10 µM norgestimate (C) demonstrates equipotent suppression of both channels. N denotes the number of tested cells. Concentration-response relationship of the inhibition of TRPC6 by norgestimate (D). Means ± SEM of n≥3 experiments per concentration are shown. The line represents the best fit of the data to the dose-response equation with a Hill slope of 1.19.

To directly investigate the effect of norgestimate on TRPC currents we performed patch clamp experiments using the same TRPC expressing cell lines used for Ca^2+^ measurements. These experiments confirmed that 10 µM norgestimate almost completely inhibited TRPC3 and −6 (**Figure 2A–C**). Further evaluation of the dose dependence of TRPC6 current inhibition by norgestimate yielded an IC_50_ of 3.06 µM (**Figure 2D**). Thus, both direct current measurements and indirect fluorescence experiments showed a similar inhibitory activity of norgestimate towards TRPC3 and −6.

Steroid-sensitivity had previously been established for TRPC5 [19] which together with its close homolog TRPC4 defines a distinct subclass of TRPC channels that is not activated by diacylglycerol. We wanted to test the subtype selectivity of norgestimate and, therefore, studied the effect of the compound on TRPC5.

**Figure 3:**
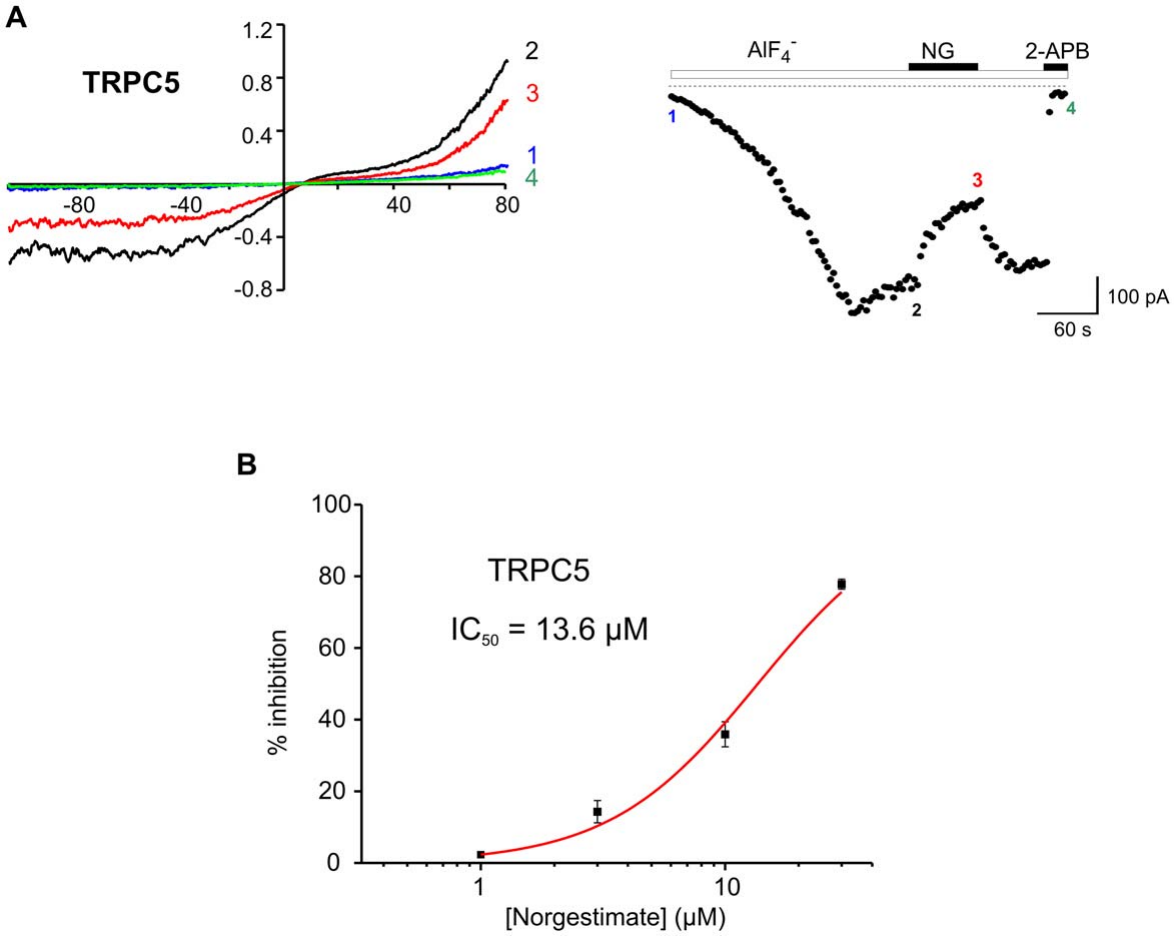
Effect of norgestimate on TRPC5-mediated currents. Whole-cell currents evoked by AlF_4_^−^ infusion into TRPC5 expressing HEK-FITR cells were measured before and after application of 10 µM norgestimate (NG) (**A**). Current-voltage (I–V) relationships (left panels) and time course of currents recorded at −70 mV (right panels) are shown. For measurement of I–V curves voltage ramps from −100 to +80 mV were applied at the time points indicated. Background currents were isolated by blocking of TRPC5 with 10 µM 2-aminoethoxydiphenyl borate (2-APB). Concentration-response relationship of the inhibition of TRPC5 by norgestimate (B). Means ± SEM of n≥3 experiments per concentration are shown. The line represents the best fit of the data to the dose-response equation with a Hill slope of 1.43.

As illustrated in **Figure 3** norgestimate suppressed TRPC5-mediated currents with an IC_50_ of 13.6 µM, indicating a considerably lower potency of the synthetic gestagen towards this diacylglycerol-insensitive TRPC channel. Unfortunately, we were unable to determine the effects of norgestimate on TRPC4 currents since compound effects at 10 µM were apparently small, irreversible and could not be separated from the significant run-down of TRPC4-mediated currents.

**Figure 4:**
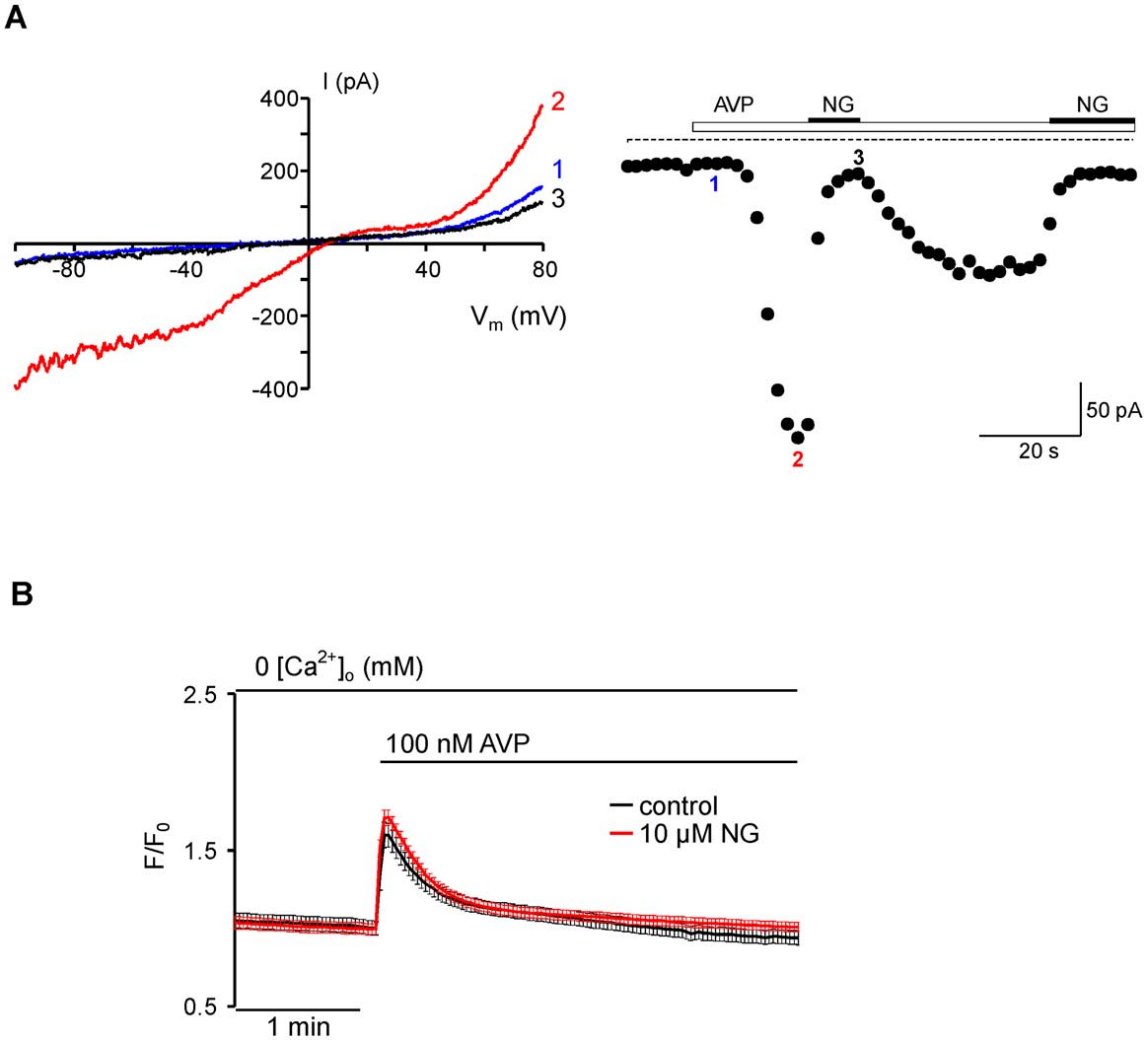
Norgestimate blocks AVP-activated non-selective cation currents in A7r5 cells independent of vasopressin receptor function. Effect of 10 µM norgestimate (NG) on whole-cell currents evoked by 100 nM AVP in A7r5 cells (**A**). I–V relationships recorded at the indicated times (left panels) and time course of currents recorded at −60 mV (right panels) are shown. The I–V curves were obtained during voltage ramps from −100 to +80 mV. Voltage-gated L-type Ca^2+^ channels were blocked by 5 µM nimodipine during the whole experiment. Time-dependent changes of [Ca^2+^]_i_ in fura-2-loaded A7r5 cells (**B**). Cells were pre-incubated with or without (control) 10 µM norgestimate (NG) in calcium-free (1 mM EGTA) standard extracellular solution for 5 min before vasopressin receptor stimulation by application of 100 nM AVP. Data represent means ± SEM from 33 cells (control) and 36 cells (norgestimate).

Having established the prominent inhibitory effect of norgestimate on recombinant TRPC6 we next asked whether the compound similarly affects native TRPC6-mediated currents. In the aortic smooth muscle cell line A7r5 TRPC6 and/or TRPC6/7 channel complexes had previously been demonstrated to underlie vasopressin V_1a_-receptor-activated cation currents [23]–[25]. Hence, we chose this model and measured inhibition of AVP-induced receptor-operated currents by norgestimate (**Figure 4A**). When 10 µM norgestimate was applied to AVP-stimulated A7r5 cells, receptor-operated cation currents measured at −70 mV were reversibly reduced by 86.5±6.0% (n = 8), in good agreement with the effect on recombinant TRPC6 channels. Since AVP-induced Ca^2+^ release was not sensitive to norgestimate (**Figure 4B**), receptor-operated channel inhibition is likely due to a direct effect on the channels proteins rather than interaction of the compound with upstream signaling elements.

**Figure 5:**
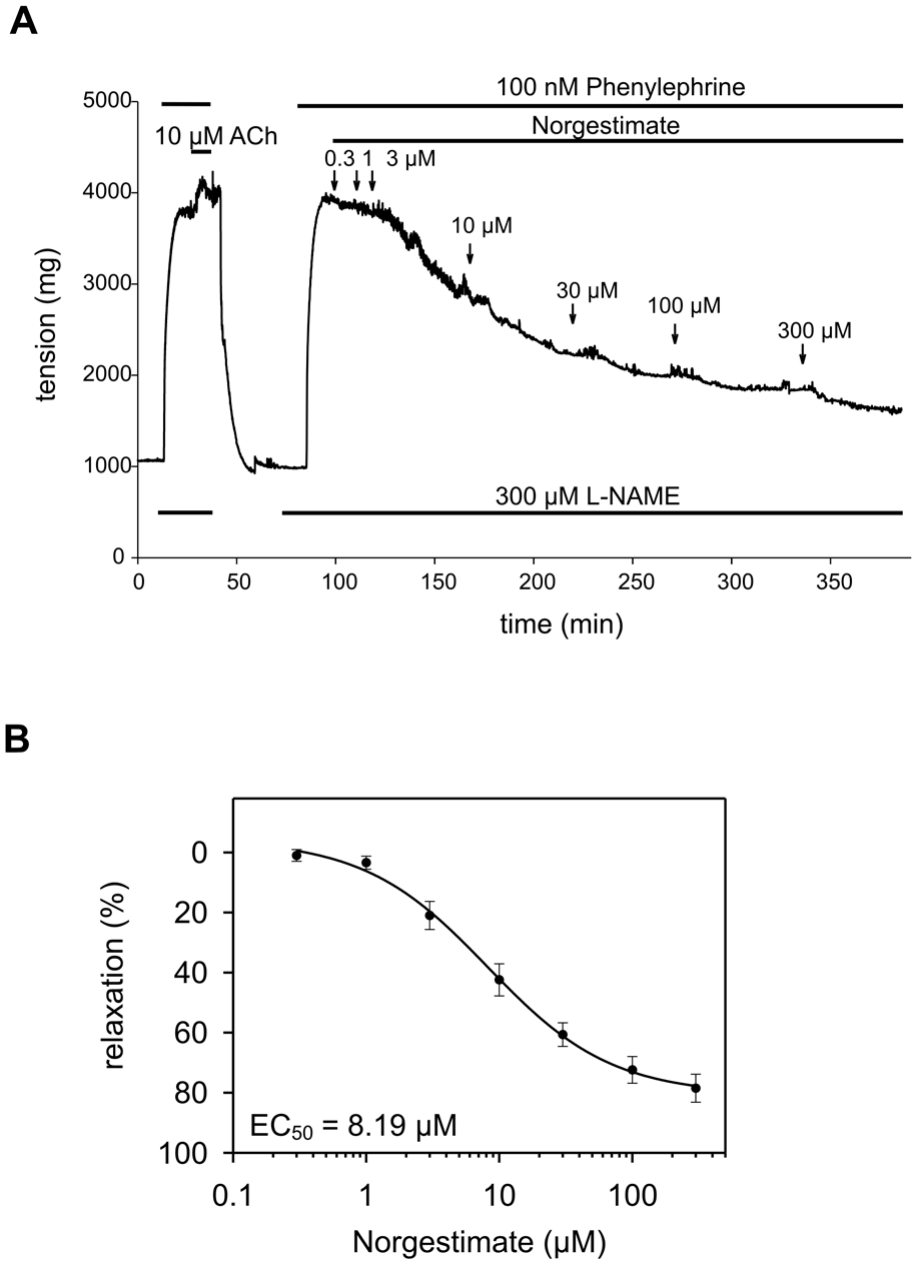
Endothelium-independent relaxation of pre-contracted rat aortic rings by norgestimate. Representative wire myograph recording (**A**) illustrating the effect of norgestimate on L-NAME treated intact aortic rings pre-contracted with phenylephrine. Compounds were applied as indicated in the perfusate. Acetylcholine (Ach) was applied to demonstrate the absence of endothelium-dependent vasorelaxation. Higher norgestimate concentrations could not be tested due to the limited solubility of the compound. Concentration-response curve of norgestimate-induced vasorelaxation (**B**). Norgestimate-induced relaxation was expressed as percentage of the phenylephrine-induced tension prior to norgestimate application. The solid line represents the best fit of the data to the Hill model with: y_0_ = −4.26%, y_1_ = 84.8%, and n = 0.93. Data represent means ± SEM (n = 6).

We extended the study of functional effects of norgestimate to intact vessel segments derived from thoracic rat aorta. Several investigators have reported expression of TRPC3/6 in this preparation [5], [26]. Isometric force measurement showed that norgestimate concentration-dependently relaxed aortic segments pre-contracted with the α_1_-receptor agonist phenylephrine (**Figure 5**). To exclude the possibility that norgestimate-induced relaxation was due to direct receptor blockage we tested the effect of the compound on recombinant α_1_-receptors expressed in CHO cells. Surprisingly, we observed that norgestimate significantly stimulated phenylephrine-induced Ca^2+^ release in this model (**Figure S1**). This stimulatory action on α_1_-receptor signaling may contribute to the relatively low vasorelaxing activity of norgestimate in isolated aortic rings. Nonetheless, our results indicate that norgestimate can be used to modulate TRPC-dependent functions *in situ*.

**Figure 6:**
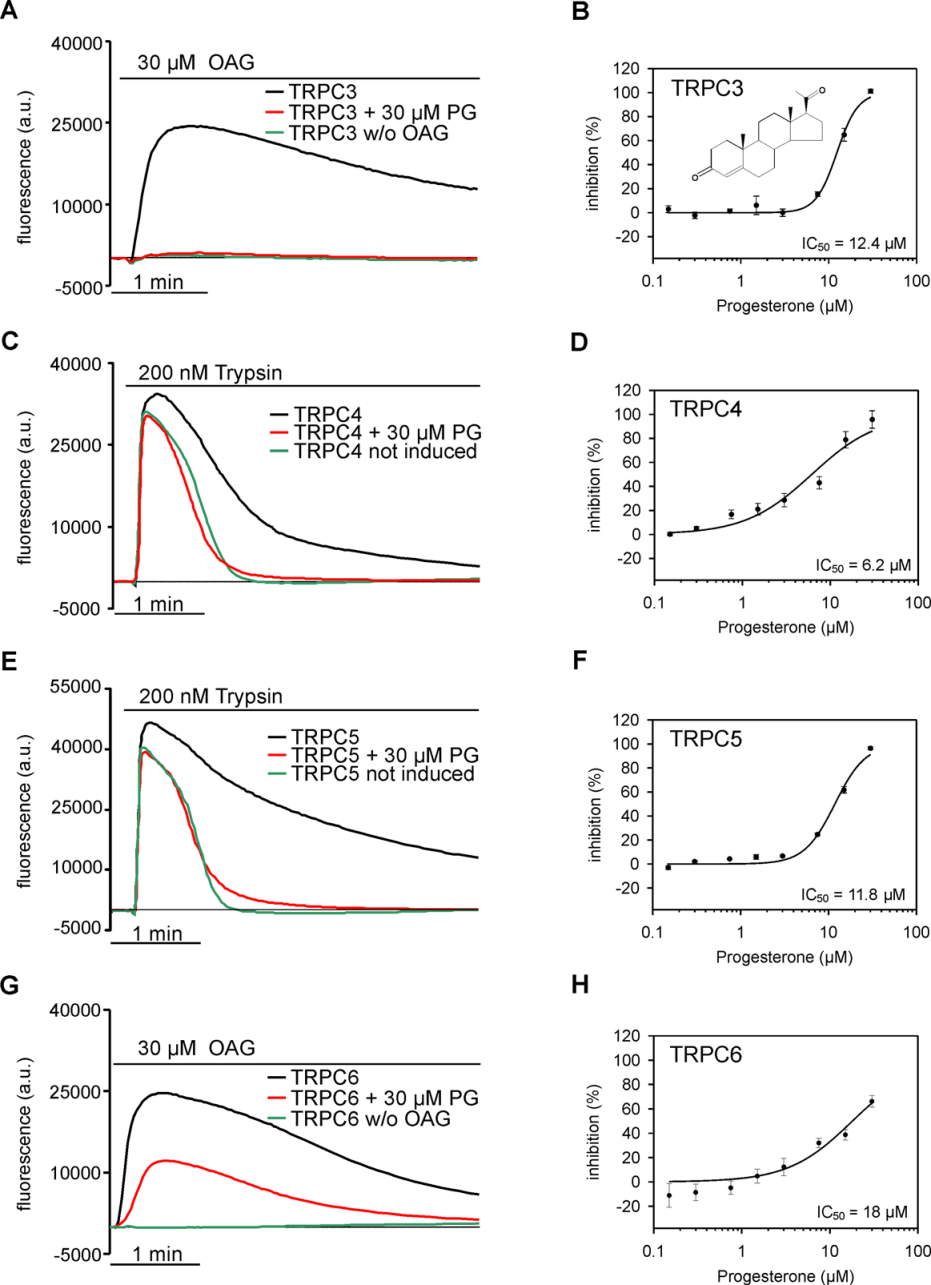
Progesterone inhibits TRPC-mediated Ca^2+^ influx. Time-dependent changes of [Ca^2+^]_i_ in fluo-4-loaded TRPC3 CHO cells (**A**), TRPC4- (**C**), TRPC5- (**E**) and TRPC6 HEK-FITR (**G**) cells were measured using a fluorometric imaging plate reader. Representative traces illustrate fluorescence changes induced by application of 200 nM trypsin or 30 µM OAG with or without pre-incubation with 30 µM progesterone (PG). TRPC-independent Ca^2+^ release in **C** and **E** was determined in non-induced TRPC4 and −5 HEK-FITR cells. Concentration response curves for inhibition of TRPC3 (**B**), TRPC4 (**D**), TRPC5 (**F**) and TRPC6 (**H**) by progesterone were derived from the area under the fluorescence curves for each given concentration. The solid lines represent the best fit of the data to the Hill model with slopes of n = 3.6 (TRPC3), 1.15 (TRPC4), 2.5 (TRPC5), and 1.2 (TRPC6). Data represent means of 3 wells (**B**) or 4 wells (**D, F, H**). The chemical structure of progesterone is illustrated in **B.**

Recent experiments revealed that TRPC5 is inhibited by certain steroids including progesterone in a stereo-selective manner [19]. Because progesterone is known to induce vasorelaxation of diverse vessels, we wondered if part of this nongenomic progesterone effect may be due to inhibition of vascular TRPC channels. Therefore, we investigated the effects of progesterone on recombinant TRPC3, −4, −5 and −6. **Figure 6** illustrates that progesterone indeed attenuated Ca^2+^ entry through all TRPCs tested. 12.4 µM and 18 µM progesterone were required for half maximal inhibition of OAG- induced [Ca^2+^]_i_ transients in TRPC3- and TRPC6 expressing cells. The hormone also suppressed trypsin-induced Ca^2+^ entry via TRPC4 and −5 with IC_50_s of 6.2 and 11.8 µM, respectively, demonstrating that diacylglycerol-insensitive TRPC family members were at least as sensitive to progesterone treatment as TRPC3 and −6.

These experiments confirm TRPC channels as novel targets of steroids and raise the possibility that TRPCs contribute to the vasoregulatory properties of these substances.

## Discussion

Our search for new pharmacological TRPC channel modulators led to the discovery of two steroids, namely norgestimate and progesterone, which differentially inhibited TRPC-mediated currents and Ca^2+^ influx.

Norgestimate was found to be a potent inhibitor of diacylglycerol-sensitive TRPC channels. The compound blocked TRPC6 in patch-clamp experiments with an IC_50_ of ∼3 µM and showed a similar or slightly higher inhibitory potency on TRPC3 currents and TRPC3-mediated Ca^2+^ entry, respectively. Diacylglycerol-insensitive TRPC5 currents were also reduced by norgestimate but required a considerably higher concentration (∼14 µM) for half maximal inhibition.

The effects of norgestimate on TRPC currents occurred rapidly, suggesting a direct modulation of channel proteins rather than genomic effects via steroid receptors. We could not discern a significant interference of 10 µM norgestimate with cytosolic calcium responses elicited by vasopressin receptors in A7r5 cells. However, the compound up-regulated α_1_-receptor induced Ca^2+^ release in CHO cells. Thus, further evaluation of norgestimate specificity is needed to fully estimate the potential applications and limitations of the compound as a blocker of diacylglycerol-sensitive TRPC channels. Nevertheless, the rather stimulatory effects of norgestimate on intracellular Ca^2+^ release exclude inositol 1,4,5-trisphosphate receptor antagonism or inhibition of the G_q/11_/phospholipase C signaling cascade as mechanisms of TRPC channel inhibition.

Notably, norgestimate inhibited recombinant TRPC6 channels and endogenous vasopressin receptor-activated cation currents in A7R5 cells with almost the same potency. There is evidence suggesting that the AVP-induced cation current in A7R5 is carried by TRPC6/7 heteromeric channels [25]. Based on this assumption it seems plausible, that other possible heteromers formed by diacylglycerol-sensitive TRPC proteins share a similar high sensitivity to norgestimate. Therefore, the steroid will be a useful tool for characterisation of native TRPC3/6/7 containing channel complexes. In line with this view, our experiments on isolated rat aorta indicate that norgestimate can be used to investigate TRPC-mediated functions in tissue preparations.

Norgestimate is a progestin (a synthetic gestagen). Combined with ethinyl estradiol it is a component of oral contraceptives widely used in humans. It would be of great interest to investigate whether other synthetic gestagens share the TRPC-blocking activity of norgestimate and whether any of the numerous cardiovascular effects of norgestimate- or other progestin-containing oral contraceptives can be assigned to TRP channel inhibition.

Prior to this study, Maheed et al. [19] showed that TRPC5 is inhibited by the pregnancy-maintaining hormone progesterone and other neurosteroids. Our Ca^2+^ measurements extent these findings and demonstrate that in addition to TRPC5 progesterone also inhibited TRPC3, −4, and −6. However, in contrast to norgestimate no preferential effects of progesterone on diacylglycerol-sensitive channel subtypes were observed. This differential sensitivity of TRPC family members to progesterone and norgestimate provides further support to the notion that channel-steroid interaction is based on direct, structure-driven binding and not caused by unspecific steroid-induced perturbation of membrane lipid bilayers.

The physiological significance of TRPC channel regulation by progesterone remains to be established. Our data suggest that in addition to neurological effects of neurosteroids [19] TRPCs may contribute to vascular effects of steroid hormones. Several studies have shown that progesterone rapidly relaxed vessels, e.g. pig coronary arteries [27] and rat aorta [28], [29]. This vasorelaxation is endothelium-independent and mediated at least partly through inhibition of L-type Ca^2+^ channels [30], [31]. Nevertheless, an involvement of other ion channels, including receptor-operated cation channels, has also been proposed [28], [29] and would be compatible with the widespread expression of TRPC channels in smooth muscle (reviewed in [32], [33]).

It is of note, that several TRPC channel proteins, in particular TRPC1, −4, and −6 have been detected in human myometrium [34]–[36]. Pregnancy is characterized by elevated progesterone plasma levels that can reach up to 1 µM [31], [37]. Given that progesterone is highly lipophilic and has a large volume of distribution [38], tissue concentrations are likely even higher and may achieve the effective concentrations for TRPC channel inhibition. Therefore, it is conceivable that TRPC channel blockade by high gestational progesterone concentrations is part of the adaptive process that limits uterine contractility during pregnancy [39].

With estimated 15 µM [40], progesterone concentrations within the placenta are even higher than in other tissues. Such levels of progesterone have demonstrated immuno-suppressive effects important for prevention of fetal-maternal rejection *in utero* [41]. As many ion channels including TRPCs are involved in immunomodulation [42] attenuation of TRPC-dependent Ca^2+^ signaling could play a role in progesterone-induced immunosuppression.

To further corroborate these hypotheses studies of TRPC channel regulation by steroid hormones in native tissues are highly desirable.

## Supporting Information

Figure S1 **Stimulatory effect of norgestimate on α_1A_- receptor-mediated Ca^2+^ signaling in CHO cells.** (**A**) Phenylephrine (Phe) -induced changes of [Ca^2+^]_i_ were measured in a stable CHO cell line expressing human α_1A_-adrenoceptors. Cells loaded with fluo-4 were pre-incubated with or without 30 µM norgestimate (NG) and challenged with 10 µM phenylephrine or control buffer. Representative traces are shown. Fluorometric imaging plate reader [Ca^2+^]_i_ measurements were performed essentially as described for HEK-FITR cells (see Materials and Methods) with the exception that the extracellular buffer contained 1 mM EGTA instead of 2 mM Ca^2+^. (**B**) The relative increase in Ca^2+^ release induced in the presence of different concentrations of norgestimate is shown. Release was estimated from the area under the curve after application of phenylephrine and normalized to the effect in the absence of norgestimate. Data shown represent means ± SEM (n = 4). Significance of changes vs. control is indicated by *(p<0.05), ** (p<0.01), and *** (p<0.001).
(TIF)
