# Supplementary figures and images for: Inhibition of Diacylglycerol–Sensitive TRPC Channels by Synthetic and Natural Steroids
Source: PLoS One. 2012 Apr 17;7(4):e35393. doi: 10.1371/journal.pone.0035393 (PMC3328449; doi:10.1371/journal.pone.0035393)

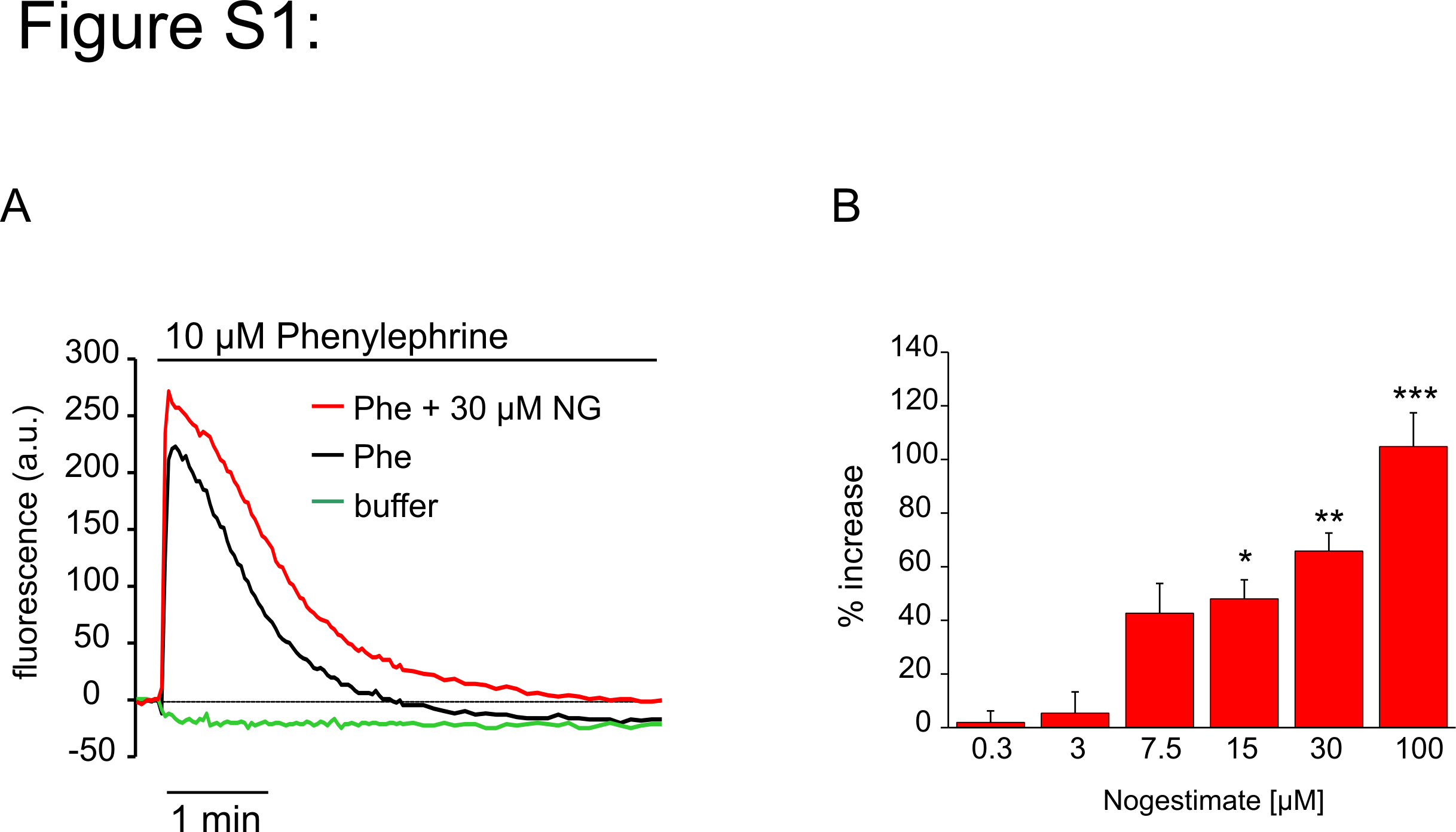

Supplement: Figure S1 — Stimulatory effect of norgestimate on α1A- receptor-mediated Ca2+ signaling in CHO cells. (A) Phenylephrine (Phe) -induced changes of [Ca2+]i were measured in a stable CHO cell line expressing human α1A-adrenoceptors. Cells loaded with fluo-4 were pre-incubated with or without 30 µM norgestimate (NG) and challenged with 10 µM phenylephrine or control buffer. Representative traces are shown. Fluorometric imaging plate reader [Ca2+]i measurements were performed essentially as described for HEK-FITR cells (see Materials and Methods) with the exception that the extracellular buffer contained 1 mM EGTA instead of 2 mM Ca2+. (B) The relative increase in Ca2+ release induced in the presence of different concentrations of norgestimate is shown. Release was estimated from the area under the curve after application of phenylephrine and normalized to the effect in the absence of norgestimate. Data shown represent means ± SEM (n = 4). Significance of changes vs. control is indicated by *(p<0.05), ** (p<0.01), and *** (p<0.001). (TIF) [file pone.0035393.s001.tif]
